# A Case of Ulceration of the Lungs

**Published:** 1892-03

**Authors:** G. J. Waggoner

**Affiliations:** Kansas City, Kansas


### A CASE OF ULCERATION OF THE LUNGS.

G. J. Waggoner, M. D., Kansas City, Kansas.

July 24th, was requested by my partner, Dr. H. A. Millard, to see Mr. L. M. A., aet. thirty-two years. He had been an invalid six months or more, it appears. It is hardly necessary to go into a history of the case for the purpose of this paper, further than to say that the disease had been supposed to be a severe form of chronic hepatitis, and although there had been some rather troublesome cough, attention had not been called to the condition of the lungs. There had been little or no expectoration, and all the functions performing their duty fairly well. On the occasion of my visit it was discovered that there was sad havoc in the lungs. On the front aspect of thorax there was no respiratory action, while on the dorsal portion there was very violent action, the back and shoulders heaving upwards and forwards, seemingly with strong but futile effort to fill the lungs. Above the lower third of left lung, in front, there was no vesicular respiration, and near the apex and just below it there was found all the evidences of a quite extensive cavity. This is well described by Dr. Thos. Watson, late of Kings College, London. In speaking of the sounds he says: "It may be, and often is a click, like the opening and shutting of a valve; or a chirp; or a creaking; or like many other well-known sounds; but as all these sounds under certain circumstances denote the formation of a vomica, it is best for the sake of simplicity to call them all by the same name, cavernous respiration." Over the front aspect of the right lung there was scarcely any vesicular murmur, and that only of the lower half. On the dorsal aspect these sounds were clear and distinct, and apparently natural. The irritation to cough was persistently felt in the stomach, which was relieved at this time and for some days later by a dose of Ant-crud.$^{1m}$. Later on there was very distressing dyspnœa, with inability to lie on left side, which was measurably relieved by a dose of Phos. But on the 9th inst. he died of strangulation.

Question: Could this result have been averted by a timely knowledge and consideration of the condition of the lungs? or, in other words, an intimate knowledge of the pathological condition? or the simillimum be considered only? An autopsy was not had, which I regret, but the patient was not mine.

